# Improvement in Hardness and Wear Behaviour of Iron-Based Mn–Cu–Sn Matrix for Sintered Diamond Tools by Dispersion Strengthening

**DOI:** 10.3390/ma14071774

**Published:** 2021-04-03

**Authors:** Elżbieta Cygan-Bączek, Piotr Wyżga, Sławomir Cygan, Piotr Bała, Andrzej Romański

**Affiliations:** 1 Center of Advanced Manufacturing Technology, Łukasiewicz Research Network—Krakow Institute of Technology, Zakopiańska 73 Str., 30-418 Krakow, Poland; piotr.wyzga@gmail.com (P.W.); slawekcyg@gmail.com (S.C.); 2 Faculty of Metals Engineering and Industrial Computer Science, AGH University of Science and Technology, 30 Mickiewicz Avenue, 30-059 Krakow, Poland; pbala@agh.edu.pl (P.B.); aromansk@agh.edu.pl (A.R.)

**Keywords:** metal-matrix composites, sintered diamond tools, abrasion wear resistance, dispersion strengthening, critical raw materials, machining

## Abstract

The work presents the possibility of fabricating materials for use as a matrix in sintered metallic-diamond tools with increased mechanical properties and abrasion wear resistance. In this study, the effect of micro-sized SiC, Al_2_O_3_, and ZrO_2_ additives on the wear behaviour of dispersion-strengthened metal-matrix composites was investigated. The development of metal-matrix composites (based on Fe–Mn–Cu–Sn–C) reinforced with micro-sized particles is a new approach to the substitution of critical raw materials commonly used for the matrix in sintered diamond-impregnated tools used for the machining of abrasive stone and concrete. The composites were prepared using spark plasma sintering (SPS). Apparent density, microstructural features, phase composition, Young’s modulus, hardness, and abrasion wear resistance were determined. An increase in the hardness and wear resistance of the dispersion-strengthened composites as compared to the base material (Fe–Mn–Cu–Sn–C) and the commercial alloy Co-20% WC provides metallic-diamond tools with high-performance properties.

## 1. Introduction

The matrix material is a crucial element in constructing a metallic-diamond tool. Its role is to hold the diamond particles during the operation of the tool and wear at a rate appropriate to the diamond particles’ wear rate to guarantee the exposure of new sharp edges of the diamond and maintain cutting capabilities. The essential matrix components in impregnated diamond tools for cutting, drilling, and grinding natural stones, concrete, and reinforced concrete are cobalt and its alloys, mainly due to excellent retention properties with diamond crystals and adequate resistance to abrasive wear. However, cobalt’s high and unstable price has compelled us to search for alternative materials. Alloyed powders based on copper and iron bearing the trade names Cobalite [1,2], Next, and Keen [3,4,5] have been developed. Due to their fine particle size, the powders can be consolidated at temperatures lower than 900 °C under moderate pressure, and the as-consolidated materials show good impact strength and durability. Despite the relatively low cost of the raw materials, the alloyed powders’ price exceeds the price of cobalt powder by 70%. Therefore, manufacturers of diamond tools are constantly looking for new, less-expensive solutions, focusing their attention on designing metallic matrix materials based on much cheaper elemental and alloy powders that would provide materials with high mechanical and tribological properties. In recent years, a new trend has been visible, consisting of cobalt-free [6,7,8], nanocrystalline [9], and dispersion-strengthened matrix materials. Common reinforcements may be added in the form of oxides, carbides, or their mixtures. Among various types of reinforcement, both SiC and ZrO_2_ are widely used because of their excellent properties, such as high elastic modulus, high strength, excellent thermal resistance, good corrosion resistance, and availability. Zaitsev et al. [10] investigated the possibilities of modifying the Cu–Ni–Fe–Sn matrix with the following nanoparticles: W, WC, NbC, Al_2_O_3_, ZrO_2_, Si_3_N_4_, and BN; they found that diamond tools with dispersed WC and ZrO_2_ nanoparticles can remarkably improve tools’ service performance during the cutting of reinforced concrete. De Oliveira et al. [11] asserted that Fe–Cu-based bonds with the addition of 1 wt.% of SiC may be used in tools dedicated to marble cutting. Tyrała et al. [12] studied the effects of ceramic reinforcement phases such as Al_2_O_3_ and Al_4_C_3_ on the as-sintered properties of the Fe–Ni–Cu–Sn–C material. Loginov et al. [13] designed novel Fe–Co–Ni binders modified with Ti and TiH_2_ to improve mechanical properties and diamond retention. Chen et al. [14] developed a Cu–Ni–Sn matrix reinforced with Al_2_O_3_ particles as a potential substitute for cobalt to fabricate diamond-cutting tools. Previous studies clearly indicate that reinforcing the metal matrix with dispersed ceramic particles could improve the quality and efficiency of metallic-diamond tools. Our previous work [15,16] confirmed that inexpensive Fe–Mn–Cu–Sn–C material can be a promising alternative to expensive Co-20% WC alloys in some sintered diamond tools. The Fe–Mn–Cu–Sn–C composites obtained by SPS (at 900 °C/35 MPa) had a hardness of 319 ± 32 HV1, high bending strength (σTRS > 1200 MPa) and bending yield strength (σ0.2 > 1000 MPa), and significantly better wear resistance than other commercial composites due to Feγ twinning and the martensitic transformation induced by plastic deformation in the sub-layer as a result of abrasion, which increases hardness and strength. The need to increase the abrasive wear resistance of these matrices arises from their application in the machining and polishing of very abrasive materials like concrete type C12/15, for which tools with a hard matrix (>110 HRB) are dedicated. The continuation of application research, as well as the further development of materials by shaping the structure and functional properties of the matrix material in metallic-diamond tools depending on the working conditions of these tools, was the essence of this research. This work proposes a new approach substituting commonly used raw materials and improving the resistance of the matrix to abrasive wear. The effects of additives such as SiC, Al_2_O_3_, or ZrO_2_ on the sintering behaviour and wear resistance of Fe–Mn–Cu–Sn–C were investigated. 

## 2. Materials and Methods

### 2.1. Materials

As a base for the matrix, a powder mixture from the Fe–Mn–Cu–Sn–C system was prepared from commercial powders. Spongy iron, ground ferromanganese, and water-atomized tin-bronze powders were provided by Höganäs AB, Höganäs, Sweden, ESAB, Katowice, Poland and Neochimie, Saint-Ouen-l’Aumône, France, respectively. The powder mixture contained 12 wt.% Mn, 6.4 wt.% Cu, 1.6 wt.% Sn, and 0.6 wt.% C and was prepared as described in [15,16]. The powders SiC (F320 series, Washington Mills Electro Minerals, Manchester, England, UK), Al_2_O_3_ (EF-320 series, Stanchem Sp.zoo, Niemce, Poland), and ZrO_2_ (Yttria-stabilized Zirconia Powder (3-YSZ) GRADE 16, HC Starck, Goslar, Germany) were used as ceramic phases to modify the material.

**Figure 1:**
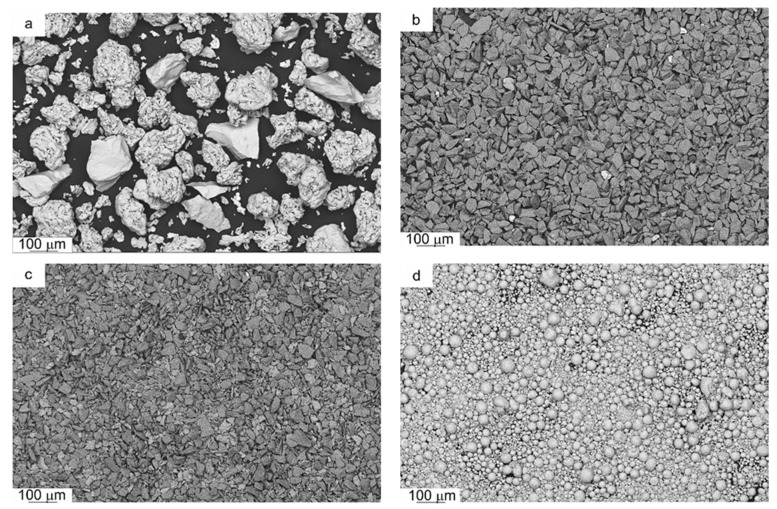
SEM images of the starting powders: (**a**) Fe–Mn–Cu–Sn–C mixture powders; (**b**) SiC; (**c**) Al_2_O_3_; (**d**) ZrO_2_.

The bulk properties of the experimental powder are presented in Table 1, and the powder particle morphology is shown in Figure 1.

In the Fe–Mn–Cu–Sn–C powder mixture, SEM images predominantly show particles with sizes above 100 µm and irregular shapes (Figure 1a). The visible fine particles of the tin bronze powder have an average particle size of 23 µm. In the case of SiC and Al_2_O_3_ powders, the SEM images show small particles with sharp edges and irregular shapes, with a size below 30 µm (Figure 1b,c). The ZrO_2_ powder is characterized by a spherical shape with an average particle size of about 40 µm (Figure 1d).

Ten mixtures were made using the ball milling method:

- Base (Fe–Mn–Cu–Sn–C);
- Base (Fe–Mn–Cu–Sn–C) with the addition of 5, 10, and 20 wt.% of SiC;
- Base (Fe–Mn–Cu–Sn–C) with the addition of 5, 10, and 20 wt.% of Al_2_O_3_;
- Base (Fe–Mn–Cu–Sn–C) with the addition of 5, 10, and 20 wt.% of the ZrO_2_.

The process of preparing the mixtures was identical for each type, and consisted of the following stages:

- Premixing of base powders from the Fe–Mn–Cu–Sn–C system for 1 h in a chaotic motion Turbula Type T2C Shaker Mixer, WAB, Muttenz, Switzerland;
- Addition of the ceramic phase in an amount of 5 wt.%, 10 wt.%, and 20 wt.% to the base mixture (Fe–Mn–Cu–Sn–C);
- Premixing of base powders (Fe–Mn–Cu–Sn–C) and ceramic phase for 1 h in a chaotic motion Turbula-type mixer;
- Ball milling in air, at 70% of the critical speed, for 8 h, with about 50 vol.% of the milling vial filled with 12 mm 100Cr6 steel balls and 10:1 ball:powder weight ratio.

### 2.2. Spark Plasma Sintering (SPS)

The powders were consolidated by SPS, employing HPD5-type equipment (manufactured by FCT Systeme GmBH, Frankenblick, Germany). The powders were placed in a graphite die (inner diameter of 20 mm), then uniaxially pressed at 35 MPa and heated to the sintering temperature at a heating rate of 100 °C/min. A 0.5 mm-thick graphite sheet was used to separate the powder from graphite die in order to prevent scuffing of the punch and facilitate extraction of the sintered sample from the die. The graphite die was also wrapped in carbon blankets to minimise heat loss during sintering. The samples were sintered in the temperature range 850–950 °C for 10 min in argon. 

### 2.3. Characterization of Sintered Specimens 

The densities of the sintered samples were measured using the Archimedes method, while their theoretical densities were calculated by applying the rule of mixtures. The hardness was determined using both the Rockwell B scale and Vickers method using a Future Tech FLC-50VX, Kawasaki, Japan hardness tester. Six indentations were made on each sample. The X-ray diffraction (XRD) patterns were obtained using a Empyrean diffractometer by PANAlytical, Malvern, UK with Cu radiation (λ_Cu_ = 1.5406 Å). The phase analysis was performed using the PANalytical High Score program integrated with the ICDD PDF-4+ crystallographic database. The Young’s moduli of the composites were determined by means of the ultrasonic wave transition method, using an ultrasonic flaw detector (Epoch III by Panametrics, Waltham, MA, USA) to measure the velocities of the ultrasonic sound waves passing through the material. The velocities of the transversal and longitudinal waves were determined as ratios of the sample thickness to the relevant transition time. 

**Figure 2:**
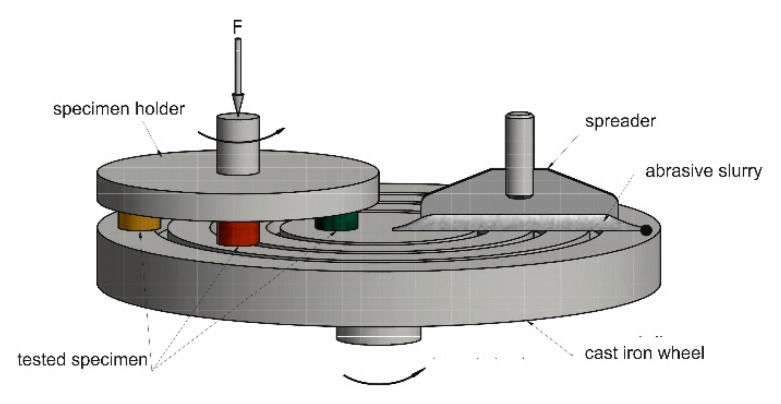
Schematic representation of the 3-body abrasive wear testing facility.

A batch of cylindrical (Ø11.3 × 5 mm) specimens was also produced for an abrasive wear test. Measurements for 3- and 2-body wear were performed using the Micro Wear Test (MWT) method [17,18,19,20] and procedure developed at AGH [20], respectively. A schematic diagram of the tester is shown in Figure 2.

**Figure 3:**
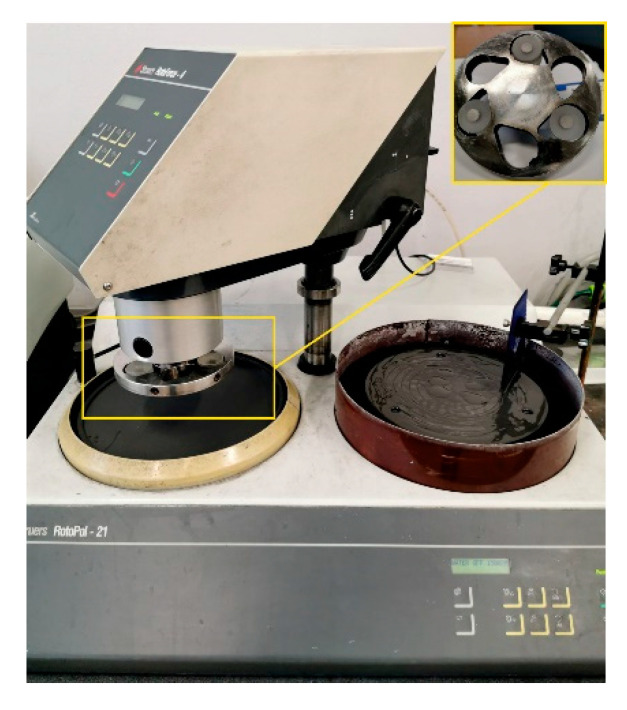
A stand for measuring of the 2- and 3-body abrasive wear resistance.

The resistance to wear in 3-body abrasion was tested simultaneously on three samples with an area of 1 ± 0.01 cm^2^ each, mounted separately in the Struers Epofix resin. The samples were positioned in a special holder and forced against an eccentrically grooved cast iron backing-wheel (∼86 HRB) under a pressure of 800 kPa. Both the backing wheel and the sample holder were set into a clockwise motion at the same rotational speed of 150 rev min^−1^. In these conditions, each tested sample was moving at a constant linear sliding velocity of 1.08 m·s^−1^. A RotoPol 21 grinder-polisher by Struers, Ballerup, Denmark equipped with a RotoForce-4 head, was used to test the abrasive wear resistance. Other accessories and fine quartz abrasive with 61% 0−0.09 mm particles and 39% 0.09−0.2 mm particles were purchased from the Danish company Fundal Consulting. The test stand is shown in Figure 3. Approximately 30 g of fine quartz abrasive (<200 μm) was suspended in 1,4-butanediol and applied to the backing wheel by means of a special spreader when the specimen holder and the backing wheel were set into a clockwise motion at the same rotational speed of 150 rev·min^−1^. This resulted in a linear sliding velocity of 1.08 m·s^−1^. The duration of a single measurement cycle was 20 s. After each cycle, the samples were cleaned in an ultrasonic cleaner in ethanol, then dried and weighed to the nearest 0.1 mg. The testing procedure required a few run-in intervals to produce a stable relief at the resin/specimen interface. As soon as the weight loss per wear interval stabilised, the abrasive index A_i3_, being inversely related to the abrasion resistance, was calculated as:

(1) $$A_{i3}=\frac{\sum \Delta M_{i}}{V\cdot A\cdot \left(\rho_{s}+2.38\right)}\cdot 10^{4},\frac{\mu m}{20m}\;,$$

where $\Delta M_{i}$ is the mass loss of an individual test piece per 20 s wear interval(g), V is the sliding velocity (m·s^−1^), A is the wear surface of the test specimen (cm^2^), and $\rho_{s}$ is the density of the test specimen (g cm^−3^).

The specimens subjected to MWT were also tested for 2-body abrasion using the same equipment. The test consisted of grinding three test pieces of each material on #220 grit SiC abrasive paper for 20 s under a pressure of 300 kPa with water as a coolant. After each test, the grinding paper was replaced with a fresh one. The loss of weight was measured for each test piece using the procedure described above, and the abrasive index A_i2_ for 2-body abrasion was calculated using Equation (1).

The microstructural studies were performed using the Axiovert 200 MAT light microscope from ZEISS, Oberkochen, Germany and the Versa 3D scanning microscope by FEI, Hillsboro, Oregon, USA (SEM) (field emission electron gun). For examination with the use of a light microscope, the samples were etched with 2% Nital. EDS examinations of selected samples were performed on non-etched specimens, and then the samples were etched in the same way as for examinations using a light microscope. The photos were taken using an SE detector. The maps were made in 50 nm steps.

## 3. Results and Discussion

**Figure 4:**
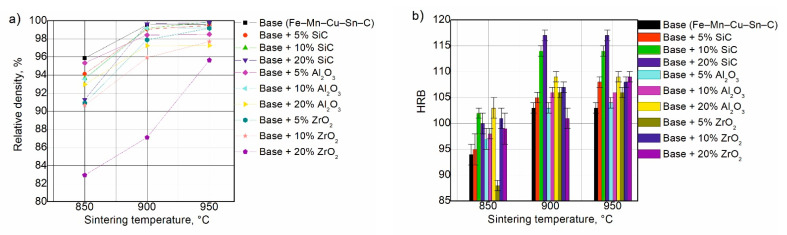
Relative density (**a**) and HRB (**b**) of iron-based matrix composites sintered by SPS.

The SPS processes were carried out in a wide range of temperatures to establish the optimal sintering conditions for all investigated materials. The relative densities and hardness numbers were used to assess the degree of consolidation of the sintered compacts. Figure 4 shows the SPS sinterability regions of the Fe–Mn–Cu–Sn–C-based composites with and without the additions.

In Figure 4, the density and hardness of the unmodified Fe–Mn–Cu–Sn–C matrix material increase with temperature up to 900 °C and do not change thereafter. The sintering behaviours in the compaction curves of the composites containing SiC and Al_2_O_3_ microparticles are similar to that of the base material. The hardness values of most composites show maxima at about 900 °C. The sintering behaviours of composites reinforced with ZrO_2_ differ from the base material and other composites. For this group of materials, acceptable density and hardness were obtained after sintering at 950 °C. The specimens with SiC additives exhibited the best mechanical properties, as hardness is the essential quality-control parameter indicating the correctness of metallic-diamond segment manufacturing processes. In the case of the tested materials, high values of this parameter were obtained for composites containing particles of the ceramic phase in 10 and 20 wt.%. Therefore, composites with 10 and 20 wt.% ceramic particles were subjected to tribological studies (Table 2).

The Young’s modulus is a quantity from which the matrix material’s retention properties (i.e., its ability to hold diamond particles) can be estimated. During the tool’s work, the diamond particles transfer variable stresses of various amplitudes to the matrix material. Under these loading conditions, the matrix should not deform plastically in order to prevent diamond particles from pulling out. Thus, to avoid plastic deformation of the matrix around the diamond particles, an ideal matrix should have a low Young’s modulus, as determined by Young’s modulus tests (Table 2). Metal-matrix composites based on Fe–Mn–Cu–Sn–C consolidated through SPS exhibited Young’s moduli from 116 to 197 GPa for materials reinforced with 20 wt.% ZrO_2_ and reference material, respectively. 

Except for the material Base + 10% ZrO_2_, which is characterized by higher wear under 2-body abrasion conditions, all the developed materials were characterized by markedly higher wear resistance for 2- and 3-body abrasion than the commonly used Co-20% WC alloy. The best results were obtained for specimens containing Al_2_O_3_ ($A_{i3}=11.0\pm 2.27$ μm/20 m, $A_{i2}=49.9\pm 11.75$ μm/20 m and $A_{i3}=11.0\pm 3.56$ μm/20 m, $A_{i2}=40.5\pm 6.4$ μm/20 m for specimens containing 10 and 20 wt.%, respectively). High wear resistance was also achieved for materials containing SiC particles ($A_{i3}=13.6\pm 2.05$μm/20 m, $A_{i2}=104.9\pm 10.62$ μm/20 m and $A_{i3}=15.5\pm 1.85$μm/20 m, $A_{i2}=$119.9 ± 10.3 µm/20 m for specimens containing 10 and 20 wt.%, respectively). The conducted research indicates that the addition of the ceramic-phase SiC and ZrO_2_ of 20 wt.% reduces the abrasive wear resistance for 2- and 3-body abrasion for SiC and for 2-body abrasion for ZrO_2_ particles compared to the base Fe–Mn–Cu–Sn–C and commercial Co-20% WC materials. Increasing the content of hard and brittle particles may change the wear mechanism from plastic to brittle (grain boundary cracking and chipping), reducing the abrasive wear resistance.

The abrasive wear resistance tests were carried out to check if the volume fraction of hard particles used in this work reduced the wear rate of the matrix. The wear rate of materials containing Al_2_O_3_ particles was the lowest, most likely due to these particles’ shape and size (21 µm). The lowest wear rate of these materials may also have been influenced by the more uniform distribution of Al_2_O_3_ in the samples’ volume and probably their better retention when compared to SiC particles. In case of SiC-containing materials, higher wear rates could be associated with SiC decomposition due to iron’s greater affinity for carbon. Thus, the diffusion of carbon to iron could take place, thereby increasing the base material’s hardness up to 160 HV1 for the material containing 20 wt.% SiC, whereas higher wear rates can probably be associated with the finer particles of SiC (compared to Al_2_O_3_), in consequence of which the softer iron-base matrix is subjected to more intensive abrasion. Further detailed research is required to determine which mechanisms are responsible for these effects and what phenomena are taking place at the metal–ceramic-particle interface.

**Figure 5:**
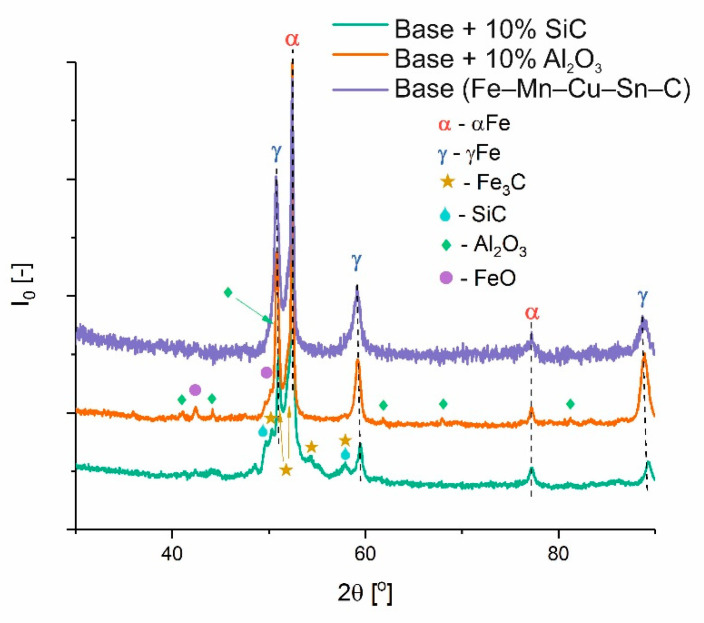
XRD analysis of iron-based composites sintered by SPS at 900 °C.

**Figure 6:**
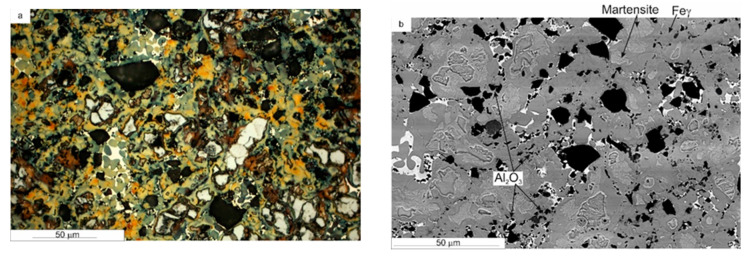

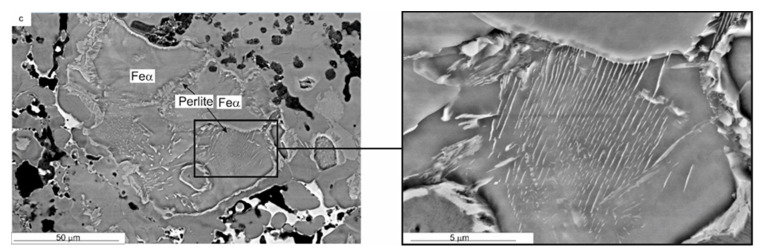
Microstructures of iron-based metal-matrix composites with the addition of 10 wt.% Al_2_O_3_ sintered by SPS at 900 °C: (**a**) LM; (**b,c**) SEM.

**Figure 7:**
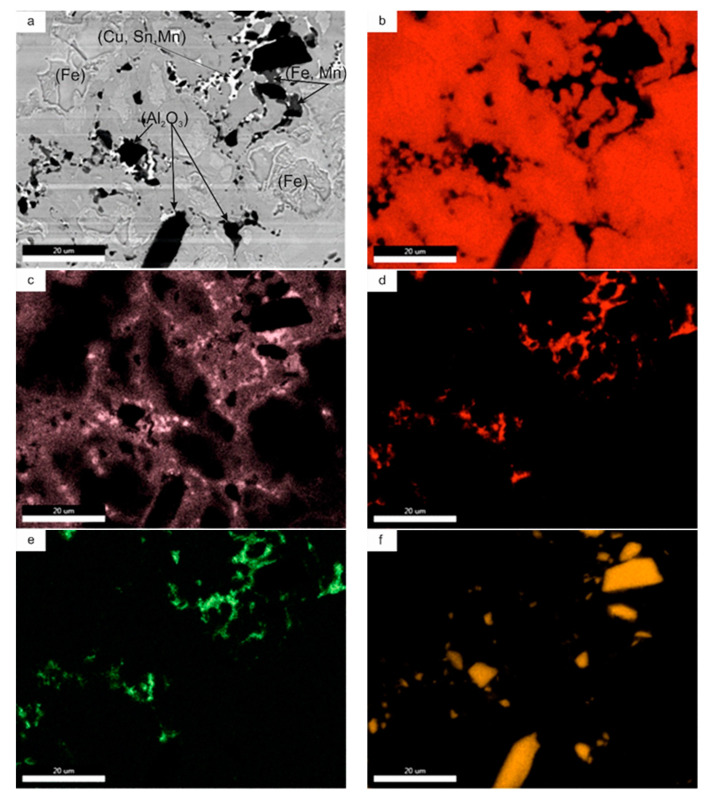

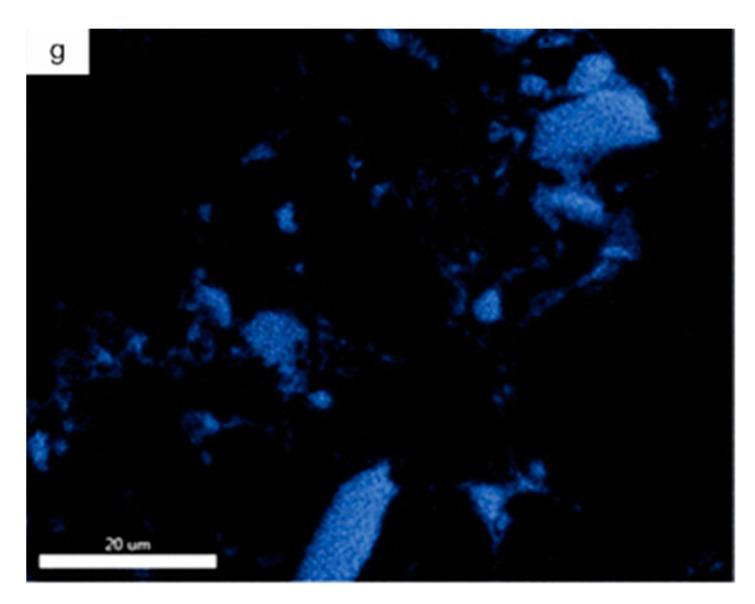
SEM image of (**a**) Base + 10% Al_2_O_3_ composite. EDS maps of: (**b**) Fe; (**c**) Mn; (**d**) Cu; (**e**) Sn; (**f**) Al; (**g**) O.

The X-ray diffraction analysis of the iron-based composites is presented in Figure 5. XRD analysis showed no other undesirable phases that could be classified as impurities. Microstructural analysis showed negligible differences in the structure of the investigated materials. The microstructures were heterogeneous, with austenitic areas enriched with Mn; areas most likely martensite (or lower bainite), perlite, ferrite, and tin bronze; and particles without any conglomerates. They were macroscopically evenly distributed. The microstructure of Base + 10% Al_2_O_3_ is exemplified in Figure 6, whereas the surface distribution of elements (EDS maps) is presented in Figure 7. It is easy to distinguish the microstructures’ constituents. The bronze area is rich in Cu, Sn, and Mn. Austenite is rich in Mn, and there is less Mn in the ferrite and martensite areas.

## 4. Conclusions

The sintering behaviours, phase compositions, microstructures, and physical and mechanical properties of iron-based matrix composites reinforced with up to 20 wt.% of SiC, Al_2_O_3_, and ZrO_2_ were investigated.

The investigated dispersion-strengthened materials based on the mixture of iron, ferromanganese, and tin bronze powders could be consolidated to near-full density by SPS for 10 min at 900 °C under a moderate pressure of 35 MPa. Dispersion-strengthened alloys showed an increase in hardness and wear resistance as compared to the base material (Fe–Mn–Cu–Sn–C) and the commercial Co-20% WC alloy.

The conducted research enables the assessment of the usefulness of ball-milled powders in the production of sintered diamond tools intended for the machining of abrasive stone and concrete.

## Figures and Tables

**Table 1:** Powder properties.

Powder	Bulk Density (g/cm^3^)	Subsieve Auto Sizer	Laser Diffraction (µm) ^1^		
		Mean Particle Size (µm)	D3	D50	D94
Base (Fe–Mn–Cu–Sn–C)	3.57	86	–		
SiC F320	1.50	31	48.98	29.97	16.53
Al_2_O_3_ EF320	1.64	21	36.3	29.1	22.6
ZrO_2_ Grade 16	1.89	43	–	0.069	0.20

^1^ Data taken from certificates.

**Table 2:** Basic physical and mechanical properties of composites sintered by SPS at 900 °C.

Material Composition (wt.%)	Density (Relative Density) (g/cm^3^)	Young’s Modulus (GPa)	HRB	HV1	$A_{i3},$ µm/20m	$A_{i2},$ µm/20m
Base (Fe–Mn–Cu–Sn–C)	7.75 ± 0.01 (>99%)	197 ± 2	103 ± 1	299 ± 7	24.6 ± 2.72	138.7 ± 1.18
Base + 10% SiC	7.27 ± 0.01 (>99%)	174 ± 2	114 ± 1	425 ± 26	13.6 ± 2.05	104.9 ± 10.62
Base + 20% SiC	6.85 ± 0.01 (>99%)	162 ± 1	117 ± 1	459 ± 31	15.5 ± 1.85	119.9 ± 10.3
Base + 10% Al_2_O_3_	7.28 ± 0.01 (99%)	194 ± 2	106 ± 1	320 ± 15	11.0 ± 2.27	49.9 ± 11.75
Base + 20% Al_2_O_3_	6.77 ± 0.01 (97%)	187 ± 2	109 ± 1	350 ± 10	11.0 ± 3.56	40.5 ± 6.4
Base + 10% ZrO_2_	7.24 ± 0.01 (96%)	172 ± 2	107 ± 1	324 ± 9	23.6± 1.35	188.9 ± 10.8
Base + 20% ZrO_2_	6.44 ± 0.01 (87%)	116 ± 1	101 ± 1	354 ± 29	28.5 ± 4.37	159.7 ± 14.8
Co-20% WC	9.26 ± 0.01 (>99%)	225 ± 3	113 ± 2	374 ± 17	48.8 ± 5.99	177.1 ± 9.23

^1^ Confidence intervals were estimated at 90% confidence level throughout the article.

## Data Availability

The data presented in this study are available on request from the corresponding author.
